# Dihydroartemisinin prevents breast cancer-induced osteolysis via inhibiting both breast caner cells and osteoclasts

**DOI:** 10.1038/srep19074

**Published:** 2016-01-08

**Authors:** Ming-Xuan Feng, Jian-Xin Hong, Qiang Wang, Yong-Yong Fan, Chi-Ting Yuan, Xin-Huan Lei, Min Zhu, An Qin, Hai-Xiao Chen, Dun Hong

**Affiliations:** 1 Orthopaedic Department, Taizhou Hospital, Wenzhou Medical University, Linhai, 317000, China; 2 Orthopaedic Department, Shanghai Key Laboratory of Orthopaedic Implant, Shanghai Ninth People’s Hospital, Shanghai Jiaotong University School of Medicine, Shanghai 200011,China; 3 Orthopaedic Department, Sir Run Run Shaw Hospital, Zhejiang University School of Medicine, Hangzhou, 310016, China

## Abstract

Bone is the most common site of distant relapse in breast cancer, leading to severe complications which dramatically affect the patients’ quality of life. It is believed that the crosstalk between metastatic breast cancer cells and osteoclasts is critical for breast cancer-induced osteolysis. In this study, the effects of dihydroartemisinin (DHA) on osteoclast formation, bone resorption, osteoblast differentiation and mineralization were initially assessed *in vitro*, followed by further investigation in a titanium-particle-induced osteolysis model *in vivo*. Based on the proved inhibitory effect of DHA on osteolysis, DHA was further applied to MDA-MB-231 breast cancer-induced mouse osteolysis model, with the underlying molecular mechanisms further investigated. Here, we verified for the first time that DHA suppressed osteoclast differentiation, F-actin ring formation and bone resorption through suppressing AKT/SRC pathways, leading to the preventive effect of DHA on titanium-particle-induced osteolysis without affecting osteoblast function. More importantly, we demonstrated that DHA inhibited breast tumor-induced osteolysis through inhibiting the proliferation, migration and invasion of MDA-MB-231 cells via modulating AKT signaling pathway. In conclusion, DHA effectively inhibited osteoclastogenesis and prevented breast cancer-induced osteolysis.

Breast cancer is one of the most common cancers among women, with bone as the most vulnerable site of distant relapse^1^. The survival rate falls from 90% for localized breast cancer to 20% for metastatic breast cancer^2^. There is a complex interplay between metastatic breast cancer cells and bone cells, known as the “vicious cycle”^3^. Breast cancer cells can express receptor activator of NF-κB ligand (RANKL), parathyroid hormone-related protein (PTHrP) and interleukins to activate osteoclast directly and indirectly, leading to enhanced osteoclastic bone resorption, which in turn release active transforming growth factor-beta (TGF-β) and other cytokines to promote breast cancer growth^3^,^4^,^5^. Currently, bisphosphonate and denosumab have been proved to be effective in treatment of breast cancer-induced osteolysis^6^,^7^. However, these therapies are still associated with side effects^8^,^9^,^10^,^11^,^12^. For instance, bisphosphonates have been shown to increase the risk of osteonecrosis of the jaw in cancer patients^3^,^13^,^14^,^15^,^16^, suggesting that other alternative treatments should be considered^17^.

Natural plant-derived compounds have received great attention because they are considered to be attractive sources of therapeutic regimens. The herb artemisia, with artemisinin as active constituent, and its derivatives, ARTs, are extensively used as anti-malarial drugs without severe side effects^18^,^19^,^20^,^21^. Dihydroartemisinin (DHA), the main active metabolite of ARTs, has been proved as the most effective anti-cancer compound^22^. DHA is capable of mediating cell cycle arrest, inducing apoptosis, blocking angiogenesis, and inhibiting metastasis^19^,^21^,^23^,^24^,^25^,^26^. More interestingly, DHA selectively kills cancer cells with little effect on normal cells^27^,^28^. Recently, DHA was found to inhibit breast cancer cells and enhance doxorubicin (DOX)-induced apoptosis^29^. However, it remains unclear whether DHA can prevent breast cancer-induced osteolysis *in vivo*. The aim of this study was to assess the effect of DHA on osteoclastogenesis and breast cancer-induced osteolysis.

## Results

### DHA inhibited RANKL-induced osteoclast differentiation and osteoclast-specific genes expression *in vitro*

**Figure 1:**
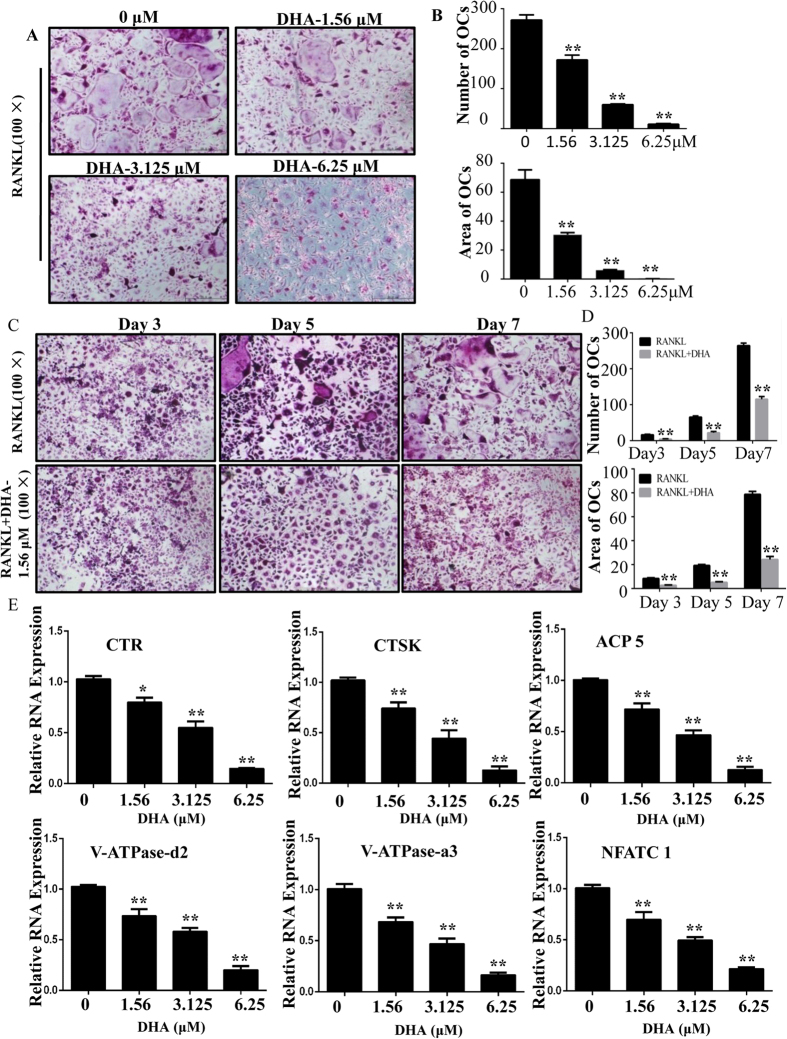
DHA inhibited RANKL-induced osteoclastogenesis and gene expression. (**A**) BMMs were treated with various concentrations of DHA, 30 ng/mL M-CSF and 50 ng/mL RANKL for 5–7 days and then subjected to TRAP staining. (**B**) The numbers and areas of osteoclasts from panel A were measured. (**C**) BMMs were treated with or without 1.56 μM DHA followed by 30 ng/mL M-CSF and 50 ng/mL RANKL for 3, 5, and 7 days and BMMs were subjected to TRAP staining. (**D**) The numbers and areas of osteoclasts from panel C were measured. (**E**) BMMs were cultured until mature osteoclasts were observed with different concentrations of DHA, 30 ng/mL M-CSF, and 50 ng/mL RANKL and then the osteoclast-specific gene expression was measured using real-time PCR. (*p < 0.05, **p < 0.01).

To investigate the effect of DHA on osteoclastogenesis, we treated primary bone marrow derived macrophages (BMMs) with RANKL and M-CSF in the absence or presence of DHA. As shown in Fig. 1A, BMMs differentiated into mature TRAP-positive multinucleated osteoclasts in the control group. In contrast, the formation of TRAP-positive osteoclasts was significantly inhibited by DHA at various concentrations (Fig. 1A,B). The number of osteoclasts (TRAP-positive with more than three nuclei) was 272.00 ± 12.77 per well in the control group and 11.00 ± 2 per well after treatment with 6.25 μM DHA. Osteoclasts were observed by TRAP staining at different time points, and osteoclast formation was significantly inhibited after 3 days of 1.56 μM DHA treatment (Fig. 1C). The number and area of osteoclasts further supported the notion that DHA could inhibit early stage osteoclastogenesis (Fig. 1D).

Based on the fact that DHA exerts inhibitory effect on osteoclast formation and function, we further explored the underlying mechanisms by which DHA regulates osteoclast differentiation and resorption. Thus, the expression of osteoclastic-specific genes was examined. In response to RANKL stimulation, osteoclast-related genes, including cathepsin k (CTSK), CTR, ACP5, V-ATPase-d2, V-ATPase-a3, and NFATc1, were up-regulated in the control group. However, a general suppressive effect on gene expression was observed in the DHA-treated group. DHA suppressed osteoclastic gene expression in a dose-dependent manner (Fig. 1E).

### DHA inhibited osteoclastogenesis without toxicity to BMMs *in vitro*

**Figure 2:**
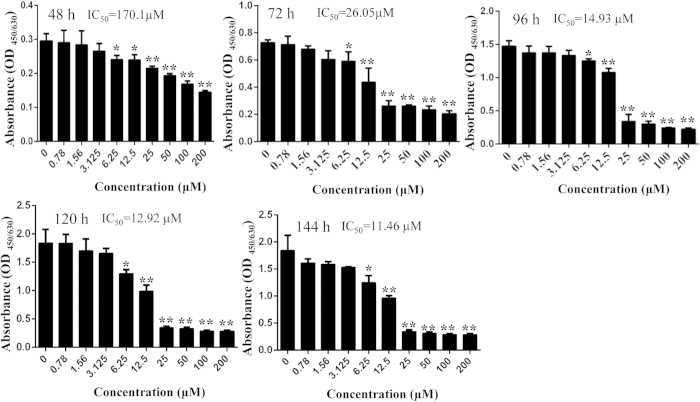
DHA inhibited osteoclastogenesis without toxicity to BMMs. BMMs were treated with indicated concentrations of DHA, 30 ng/mL M-CSF, and 50 ng/mL RANKL for 48 h, 72 h, 96 h, 120 h and 144 h, respectively, and cell viability was then measured using CCK-8 assay. The IC_50_ for DHA in BMMs at 48 h, 72 h, 96 h, 120 h and 144 h were also calculated. (*p < 0.05, **p < 0.01).

To exclude the possibility that the inhibitory effect of DHA on osteoclast formation was related to cytotoxic effect, cell viability assay was also performed. We demonstrated that cell viability was not affected by DHA at concentrations lower than 6.25 μM, at which concentration osteoclastogenesis was significantly inhibited (Fig. 2). The calculated IC_50_ for DHA in BMMs was 170.1 μM at 48 h, 26.05 μM at 72 h, 14.93 μM at 96 h, 12.92 μM at 120 h and 11.46 μM at 144 h (Fig. 2). Together, these results demonstrate that DHA suppressed osteoclast differentiation without toxicity to BMMs.

### DHA inhibited osteoclast F-actin ring formation and bone resorption *in vitro*

**Figure 3:**
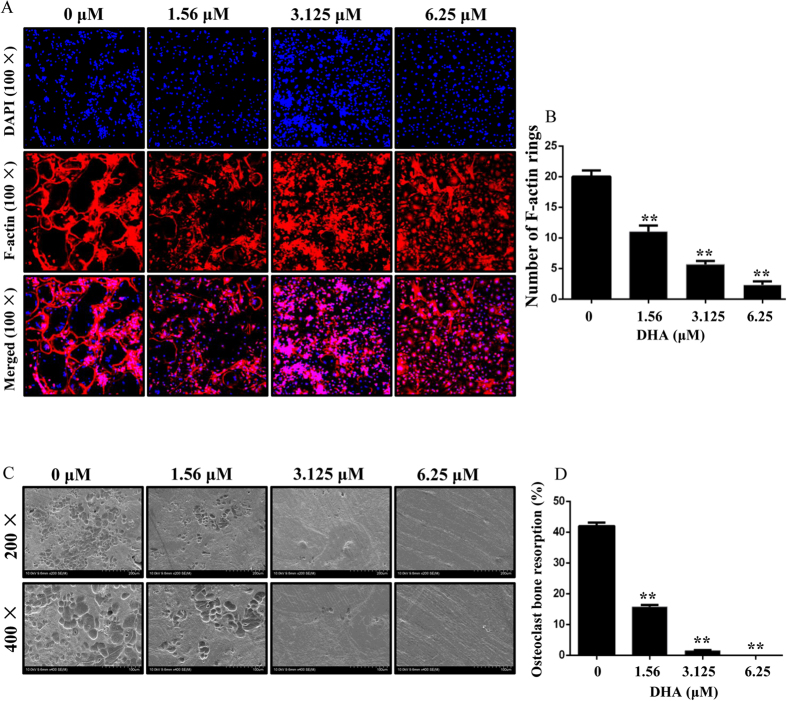
DHA inhibited F-actin ring formation and osteoclastic bone resorption activity *in vitro.* (**A**) BMMs were treated with different concentrations of DHA, 30 ng/mL M-CSF, and 50 ng/mL RANKL until osteoclasts formed. The F-actin rings and cell nuclei were stained with Alexa-Fluor 647 phalloidin and DAPI respectively and observed under a confocal microscope. (**B**) The number of intact F-actin rings was calculated. (**C**) BMMs were treated with different concentrations of DHA, 30 ng/mL M-CSF, and 50 ng/mL RANKL until mature osteoclasts formed and functioned. The imagines of bone resorption pits were shown by Scanning electron microscope (SEM). (**D**) Resorption pit areas were measured as mentioned in methods. (*p < 0.05, **p < 0.01).

The formation of a well-polarized F-actin ring is necessary for osteoclastic bone resorption, we next tested the effect of DHA on F-actin ring formation. Characteristic F-actin ring formation was observed in the control group, as visualized by Phalloidin-Alexa Fluor 647 and DAPI staining (Fig. 3A). However, treatment with DHA resulted in drastic alterations in F-actin ring number and morphology. The number of F-actin rings was 21.00 ± 1 per view in the control group, 11.00 ± 1 per view after treatment with 1.56 μM DHA, 3.67 ± 0.58 per view after treatment with 3.125 μM DHA, and 1.33 ± 0.58 per view after treatment with 6.25 μM DHA. (Figure 3B), suggesting that DHA impaired osteoclast F-actin ring formation *in vitro*.

Due to the inhibitory effect of DHA on osteoclast differentiation and F-actin ring formation, it is expected that osteoclastic bone resorption may be impaired by DHA treatment. Indeed, large bone resorption pits were observed in the control group while smaller and fewer resorption pits were observed in the DHA treated groups. The resorption area decreased to ∼20% and less than 5% after treatment with 1.56 and 3.125 μM DHA, respectively. Almost no resorption pit was observed on the slices treated with 6.25 μM DHA (Fig. 3C,D). Together, these data indicate potential inhibitory effect of DHA on osteoclastic bone resorption.

### DHA does not affect osteoblast differentiation and osteoblastic-specific genes expression *in vitro*

**Figure 4:**
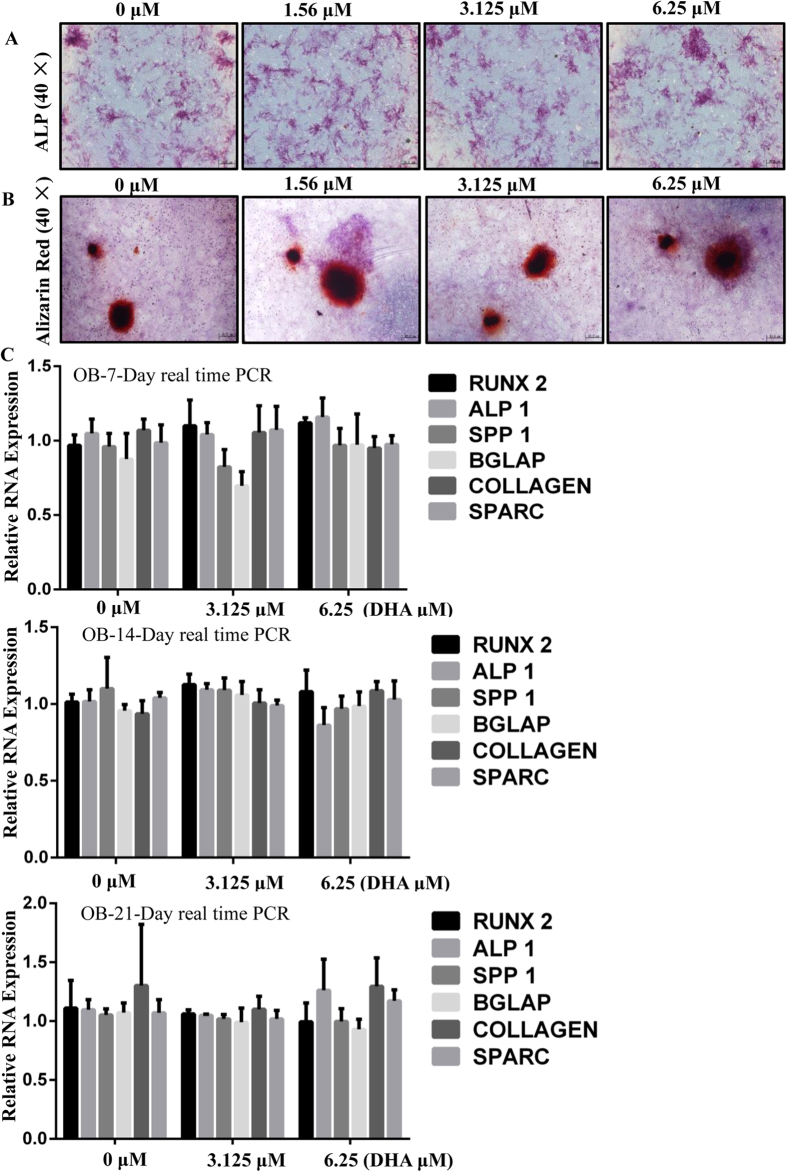
DHA does not affect osteoblast differentiation and gene expression *in vitro.* (**A**) BMSCs were were cultured in differentiation medium with the various concentrations of DHA for 7 days and the Alkaline phosphatase (ALP) staining showed no significant difference between control and DHA treated groups. (**B**) BMSCs were cultured in differentiation medium with various concentrations of DHA for 21 days and Alizarin Red Staining demonstrated no significant difference between control and DHA treated group. (**C**) BMSCs were were cultured in differentiation medium with various concentrations of DHA for 7, 14, or 21 days, respectively and the osteoblast-specific gene expression was analyzed by real-time PCR. (*p < 0.05, **p < 0.01).

Although DHA impaired osteoclast formation, it is important to ask if DHA has any effect on osteoblasts. Therefore, Bone marrow derived mesenchymal stem cells (BMSCs) were isolated for osteoblastic differentiation and mineralization. Alkaline phosphatase (ALP) staining clearly showed no significant difference between control and DHA treated groups (Fig. 4A). In addition, alizarin red staining also demonstrated that the total mineralized area of each dish was similar between the control group and DHA treated group (Fig. 4B). We further explored the expression of osteoblastic-specific genes, including RUNX 2, COL, ALPl, Bglap, SPP1, and Sparc. The mRNA expression of osteoblast differentiation markers was not affected by DHA treatment with 3.125 and 6.25 μM DHA at 7 days, 14 days, and 21 days (Fig. 4C). These results suggest that DHA does not affect osteoblast differentiation and mineralization.

### DHA suppressed titanium-particle-induced osteolysis *in vivo*

**Figure 5:**
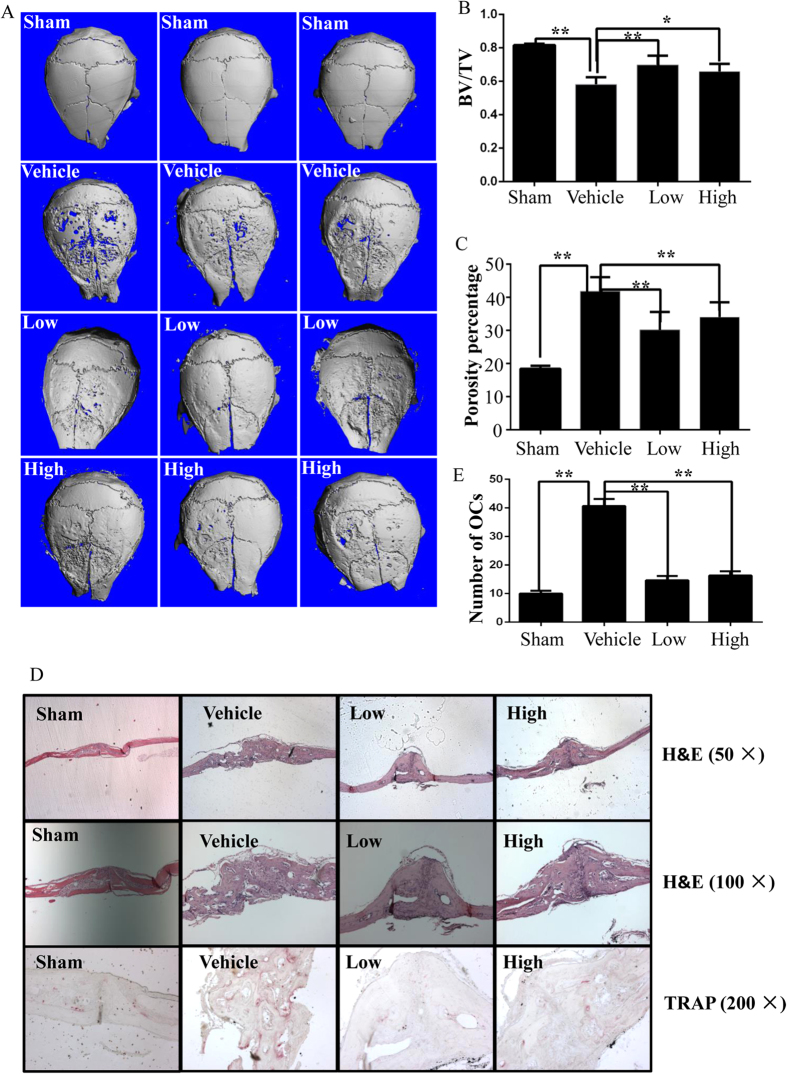
DHA inhibited titanium-particle-induced murine calvarial osteolysis *in vivo.* (**A**) Representative micro-CT and 3-dimensional reconstructed images from each group are shown. (**B**) The BV/TV of each sample was measured. (**C**) The percentage of total porosity of each sample was measured.(**D**) Calvarial sections were fixed, decalcified, dehydrated, and sectioned. Sections were stained with H&E and TRAP. (**E**) The number of osteoclasts per field of tissue (no. of OCs/field) was analyzed. (*p < 0.05, **p < 0.01).

Given the fact that DHA impaired osteoclast formation but not osteoclast differentiation *in vitro*, it is important to investigate whether DHA is capable of prevent osteoclastic osteolysis *in vivo*. Thus, the effect of DHA on titanium-particle-induced osteolysis murine calvarial model was measured. We found that extensive bone resorption was observed in the vehicle group by micro-CT. In the groups with DHA treatment, reduced particle-induced osteolysis was observed (Fig. 5A). Bone volume against tissue volume (BV/TV) and the percentage of total porosity in the ROI were measured from three dimensional reconstruction images. When DHA was injected at 50 μg/kg and 100 μg/kg daily, osteolytic bone loss was prevented. The bone loss in vehicle group (BV/TV: 0.582 ± 0.042) was significantly prevented after being treated with 50 μg/kg of DHA (BV/TV: 0.699 ± 0.054) and 100 μg/kg of DHA (BV/TV: 0.659 ± 0.045) (Fig. 5B,C).

The histological and histomorphometric analysis further confirmed the protective effect of DHA on wear particle-induced bone erosion (Fig. 5D). Multinucleated osteoclasts as well as the inflammatory infiltration of lymphocytes and macrophages were induced by the presence of Ti particles. TRAP staining revealed that multiple osteoclasts lined along the eroded bone surface in the vehicle group. The number of osteoclasts was reduced in the DHA treatment groups (Fig. 5E). This result indicates that DHA inhibited osteoclast formation and function *in vivo*.

### DHA suppressed AKT/SRC signaling pathways during osteoclastogenesis

**Figure 6:**
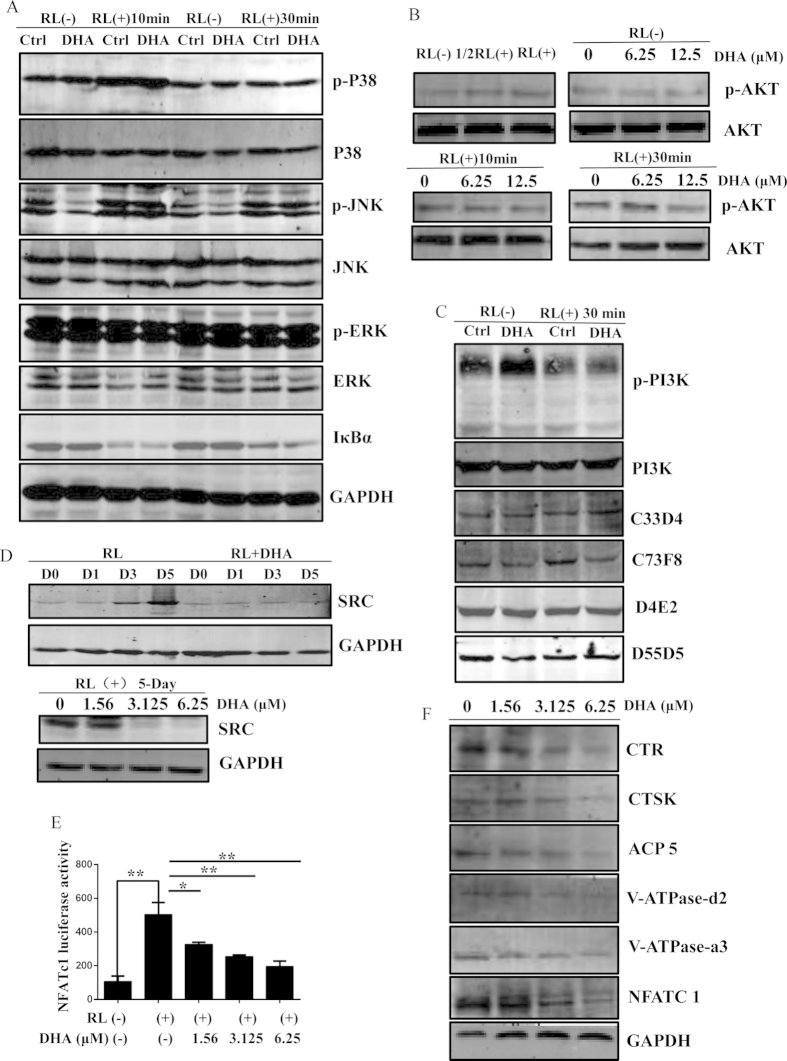
DHA specifically impaired AKT/SRC signaling pathways during osteoclastogenesis. (**A**) RAW264.7 cells were pretreated with or without 6.25 μM DHA for 4 h followed by 50 ng/mL RANKL for 0, 10 or 30 min respectively, and the cell lysates were analyzed using western blotting for MAPK and IκBα signaling pathways. (**B**) RAW264.7 cells were pretreated with various concentrations of DHA for 4 h followed by 50 ng/mL RANKL or different concentrations of RANKL for 0, 10 or 30 min respectively, and the cell lysates were analyzed using western blotting for AKT signaling pathway. (**C**) RAW264.7 cells were pretreated with or without 6.25 μM DHA for 4 h followed by 50 ng/mL RANKL for 0 or 30 min respectively, and the cell lysates were analyzed using western blotting for PI3K signaling pathway. (**D**) BMMs were cultured with or without 3.125 μM DHA, 30 ng/mL M-CSF, and 50 ng/mL RANKL for 0, 1, 3, or 5 days and the cell lysates were analyzed using western blotting for SRC signaling pathway. RAW264.7 cells were cultured with various concentrations of DHA followed by 50 ng/mL RANKL for 5 days and the cell lysates were analyzed using western blotting for SRC signaling pathway. (**E**) Luciferase cells were plated in 24-well plates and pretreated with 0, 1.56, 3.125, or 6.25 μM DHA for 1 h, and then incubated with 50 ng/mL RANKL for 24 h to activate NFATc1. Luciferase activity was detected using the Luciferase Assay Kit. (**F**) BMMs were cultured with various concentrations of DHA, 30 ng/mL M-CSF, and 50 ng/mL RANKL for for 5–7 days until osteoclasts formed and the cell lysates were analyzed using western blotting to detect the protein expression levels of CTSK, CTR, ACP5, V-ATPase-d2, V-ATPase-a3, NFATc1 and GAPDH (*p < 0.05, **p < 0.01).

To explore the potential mechanisms on how DHA affects osteoclast formation, we initially investigated the role of DHA on critical signaling pathways during osteoclast formation. NF-κB signaling pathway is one of the key signaling pathways during osteoclast formation. Thus, we investigated IκBα activation. However, there is no suppressive effect of DHA on IκBα degradation (Fig. 6A), MAPK signaling pathways are also imperative for osteoclast differentiation. The phosphorylation of p38, Erk and JNK were further investigated. However, with the treatment of DHA, there is no difference between the control and DHA treated groups, indicating no suppressive effect of DHA on these signaling pathways (Fig. 6A). In addition, PI3K-AKT signaling pathway is also important for osteoclast formation. Interestingly, we found that the phosphorylation of AKT was observed after stimulation with RANKL for 10 min and 30 min. In contrast, a significant suppressive effect of p-AKT was observed in the DHA-treated group after stimulation with RANKL for 30 min (Fig. 6B). Importantly, the activation of PI3K, the upper steam of AKT, was not affected by DHA treatment (Fig. 6C). Moreover, the increased expression of SRC during osteoclast differentiation was inhibited dramatically by DHA treatment at day 3 and day 5. The expression of SRC was significantly also inhibited in RAW264.7 cells by DHA treatment (Fig. 6D). Besides, NFATc1 luciferase activity was inhibited (Fig. 6E) and NFATc1 protein level was reduced in the DHA-treated group (Fig. 6F). Furthermore, we investigated the protein expression level of osteoclast specific gene, including CTSK, CTR, ACP5, V-ATPase-d2, V-ATPase-a3, and NFATc1. In agreement with the inhibitory effect on mRNA level, a general suppressive effect on osteoclast specific protein expression was observed in the DHA-treated group (Fig. 6F). Collectively, these data suggest that DHA may exert an inhibitory effect on AKT and SRC activation.

### DHA inhibits the proliferation, migration and invasion of MDA-MB-231 breast cancer cells *in vitro*

**Figure 7:**
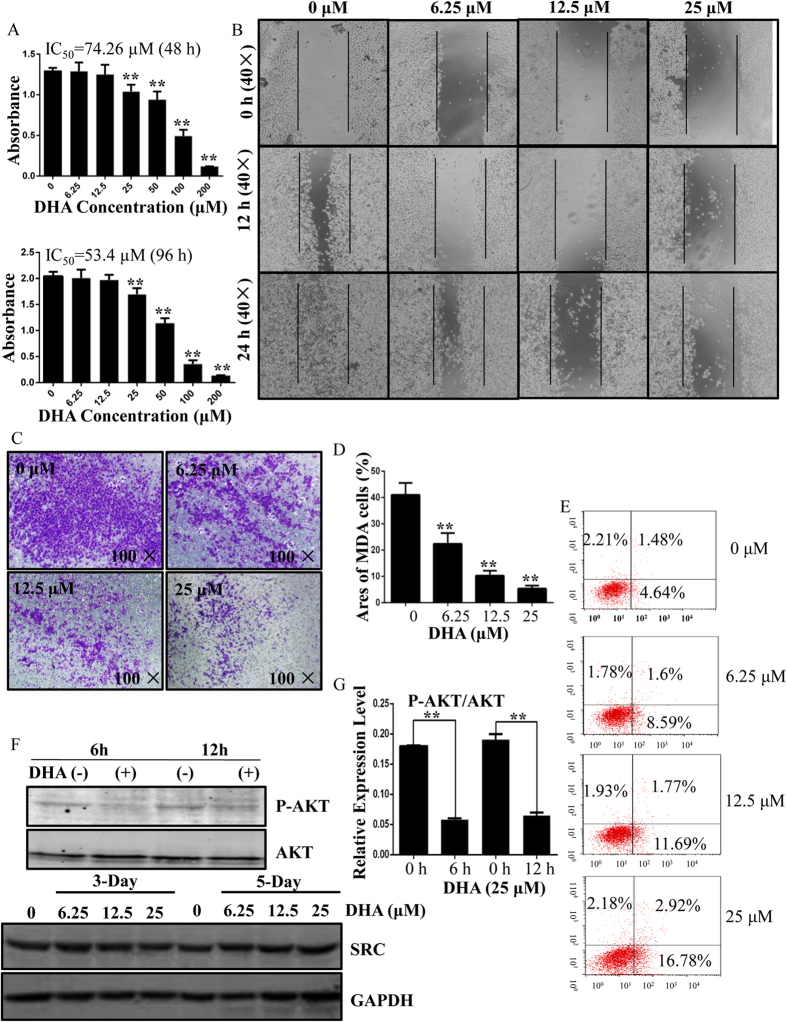
DHA inhibits the proliferation, migration, and invasion of MDA-MB-231 cells and promotes apoptosis. (**A**) Viability of DHA-treated MDA-MB-231 cells. The calculated IC50 was 74.26 μM (48 h) and 53.4 μM (96 h) respectively. (**B**) DHA inhibits monolayer wound healing of MDA-MB-231 cells. (**C**) Membrane-associated, crystal violet stained MDA-MB-231 breast cancer cells following treatment with various concentrations of DHA for 24 h. (**D**) The areas of invasive cells were counted. (**E**) Flow cytometric analysis of DHA-treated MDA-MB-231 cells. (**F**) DHA reduces phosphorylated AKT expression, but does not effect the expression of SRC. (**G**) The band intensity corresponding to phosphorylated AKT was quantified and normalized to GAPDH using Image J. (*p < 0.05, **p < 0.01).

Based on the evidence that DHA is a potential natural compound to prevent osteoclastic osteolysis, we further asked if DHA is active in preventing breast cancer-induced osteolysis. Thus, the effect of DHA on breast cancer cells was initially investigated. Following a 48 h and 96 h culture, a CCK-8 proliferation assay revealed that DHA can inhibit MDA-MB-231 cell proliferation with the calculated IC50 of 74.26 μM (48 h) and 53.4 μM (96 h) respectively (Fig. 7A). Furthermore, DHA inhibits MDA-MB-231 cell migration in a concentration-dependent manner (Fig. 7B). DHA also inhibits MDA-MB-231 cell invasion in a concentration-dependent manner. The areas of MDA-MB-231 cells were 41.03 ± 4.58%, 22.33 ± 4.16%, 10.27 ± 1.86%, 5.37 ± 1.11% when treated with 0, 6.25, 12.5, 25 μM DHA at 24 h. Quantitative analysis revealed that DHA can inhibit cell invasion at concentrations as low as 6.25 μM (Fig. 7C,D). In addition, we noticed that DHA at 6.25, 12.5 or 25 μM induced different degree of apoptosis, with about 16.78% of apoptotic cells at the concentration of 25 μM (Fig. 7E). As we found DHA suppressed AKT phosphorylation during osteoclastogenesis, it is worth of checking whether DHA also modulate AKT signaling pathway in breast cancer cells. Upon 6 h and 12 h DHA treatment on breast cancer cells, the AKT phosphorylation was significantly inhibited (Fig. 7F,G). This result indicates that DHA inhibited AKT activation in MDA-MB-231 cells.

### DHA inhibits breast cancer bone metastasis and osteolysis *in vivo*

**Figure 8:**
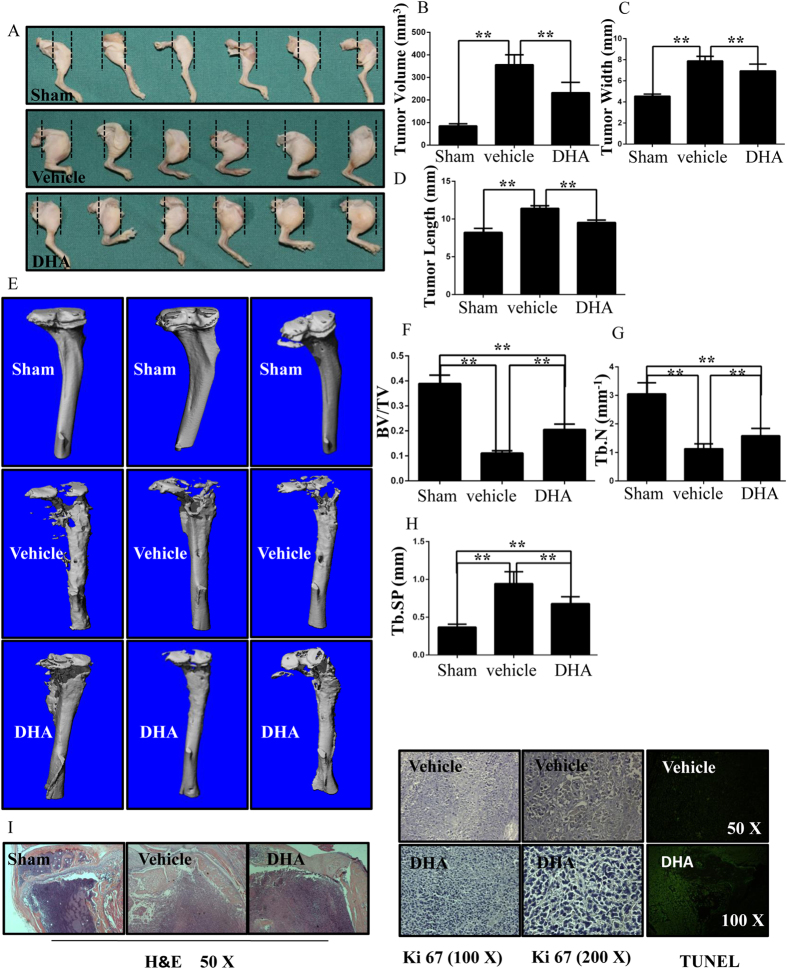
DHA inhibits breast cancer bone metastasis and osteolysis *in vivo.* (**A**) MDA-MB-231 cells were injected directly into the tibiae plateau. After 28 days, the tissue volume (**B**), tissue width (**C**) and tissue length (**D**) were measured in the vehicle group. (**E**) Representative micro-CT and 3-dimensional reconstructed images from each group are shown. (**F**) The BV/TV of each sample was measured. (**G**) Trabecular number (Tb.N) of each sample was measured. (**H**) Trabecular separation (Tb.Sp) of each sample was measured. (**I**) The tumor volume sections were fixed, decalcified, dehydrated, and sectioned. Sections were stained with H&E, Ki67 and TUNEL. (*p < 0.05, **p < 0.01).

To determine the effect of DHA on breast cancer-induced osteolysis *in vivo*, a mouse xenotransplant model was used. MDA-MB-231 cells were injected directly into the tibiae plateau via a percutaneous approach. After 28 days, the tissue volume, tissue length and tissue width were reduced in the DHA group compared with those in the vehicle group (Fig. 8A), indicating that DHA effectively suppressed breast cancer bone metastasis and growth *in vivo*. These observations were consistent with the results of the tumor volume assay (Fig. 8B–D). The tumor volume in vehicle group (355.98 ± 44.22 mm^3^) was significantly higher than that in the DHA (100 μg/kg) group (232.05 ± 46.08 mm^3^). To confirm that osteolytic bone metastasis was inhibited by DHA, the osteolysis region in the long bones of the hind legs was examined using micro-CT (Fig. 8E). The BV/TV in vehicle group (0.111 ± 0.009) was significantly lower in comparison with that in the sham group (0.389 ± 0.034). In contrast, the BV/TV in the DHA (100 μg/kg) group was 0.205 ± 0.022 (Fig. 8F). In addition, the administration of DHA produced a higher trabecular number (Tb.N) and reduced trabecular separation (Tb.Sp) than the vehicle group (Fig. 8G,H). The apoptosis TUNEL assay was also performed. The levels of apoptosis were significantly increased in the DHA-treated group compared with the vehicle group (Fig. 8I). All *in vivo* data demonstrated that DHA inhibited MDA-MB-231 cancer cell-induced osteolysis.

## Discussion

Bone metastasis is a critical complication in patients with advanced breast cancer, leading to severe pain, pathological fracture and hypercalcemia, all of which adversely affect the patients’ quality of life and survival^6^,^30^,^31^,^32^. Although the molecular mechanism underlying the preferential metastasis of breast cancer to bone is yet to be elucidated, it is believed that the crosstalk between metastatic breast cancer cells and osteoclasts is critical for osteolysis^3^,^4^,^5^,^33^.

In this study, we have verified for the first time that DHA suppressed osteoclast differentiation in a dose-dependent manner without cytotoxic effect to BMMs, which was further supported by the fact that DHA suppressed osteoclastic specific gene and protein expression in a dose-dependent manner. We also demonstrated that the F-actin ring formation, a prerequisite for efficient bone resorption^34^, was significantly inhibited by DHA. Consequently, DHA impaired osteoclastic bone resorption in a dose-dependent manner. Based on the fact that DHA suppressed osteoclast formation and function *in vitro*, we further proved that DHA prevented wear particles induced osteolysis *in vivo*, supported by both reduced bone resorption area and reduced TRAP positive osteoclasts. Importantly, we also found that DHA does not affect osteoblast differentiation and osteoblast related gene expression, suggesting DHA is an attractive natural compound for osteolytic bone diseases.

Recently, DHA was reported to inhibit breast cancer cells and enhance doxorubicin (DOX)-induced breast cancer cell apoptosis^29^. However, it remains unclear whether DHA can prevent breast cancer-induced osteolysis *in vivo*. Therefore, we further investigated the role of DHA on a breast cancer-induced osteolysis model. Interestingly, DHA suppressed tumor growth and protected breast cancer-induced osteolysis *in vivo*. On one hand, the suppressive effect is due to the inhibitory effect of DHA on the proliferation, migration and invasion of MDA-MB-231 cells. On the other hand, this effect may also due to the fact that DHA can inhibit osteoclastic bone resorption *in vivo*, supported by the preserved bone mass in the DHA treated groups.

Accumulative evidence shows that the PI3K/AKT pathway plays an important role in osteoclast differentiation^35^,^36^,^37^. In consistent with these findings, we also demonstrated that the inhibition of AKT signaling pathway by DHA can impair osteoclast formation and function. Interestingly, we found the activation of upper stream protein PI3K was not affected by DHA treatment, indicating AKT is a potential binding target of DHA. However, further experiments on the mode of action of DHA on AKT are still required. In osteoclasts, the sequential activation of PI3K and AKT is known to involve SRC proteins. The non-receptor tyrosine kinase SRC is an important molecule in cell migration, adhesion, and osteoclast-mediated bone resorption^38^. Here in our study, we also demonstrated that the expression of SRC was inhibited in osteoclasts, suggesting DHA inhibits osteoclasts via modulating AKT/SRC signaling pathway. Furthermore, we also investigated the impact of DHA on NFATc1 which is one important downstream target of RANKL-induced osteoclastogenesis. As expected, the NFATc1 luciferase activity and the NFATc1 protein expression were significantly inhibited by DHA. Taken together, we showed that the mechanism of DHA-mediated inhibition was via the suppression of AKT/SRC signaling.

In breast cancer, PI3K/AKT/mTOR is the most frequently activated signaling pathway to promote tumor growth and progression^39^,^40^,^41^. Here, we also demonstrated that DHA can inhibit AKT activation, thus leading to inhibited proliferation and enhancing apoptosis in breast cancer cells. This finding suggests a universal mode of action of DHA is related to AKT activation. In conclusion, this study described a natural compound DHA can effectively inhibit osteoclastogenesis and prevented breast cancer-induced bone osteolysis through the suppression of the AKT signaling cascade.

## Materials and Methods

Animal care and experiments were conducted in accordance with guidelines and procedures authorized by the Animal Experimental Ethical Committee of Taizhou Hospital, Wenzhou Medical University.

### Media and reagents

DHA was purchased from Sigma-Aldrich (St Louis, MO, USA). The Alpha modification (α-MEM), dulbecco’s modified eagle medium (DMEM), fetal bovine serum (FBS), and penicillin/streptomycin were purchased from Gibco-BRL (Gaithersburg, MD, USA). The cell counting kit-8 (CCK-8) was obtained from Dojindo Molecular Technology (Tokyo, Japan). Soluble mouse macrophage colony-stimulating factor (M-CSF) and RANKL were obtained from R&D Systems (USA). The specific primary antibodies and secondary antibodies used in the experiments were obtained from Cell Signaling Technology (Cambridge, MA, USA) and Sigma-Aldrich (St Louis, MO, USA). The tartrate-resistant acid phosphatase (TRAP) staining kit, Triton X-100, and 4′,6-diamidine-2′-phenylindole dihydrochloride (DAPI) were purchased from Sigma-Aldrich. The Vybrant^®^ Apoptosis Assay kit #2 was from Invitrogen (Carlsbad, CA, USA).

### Cell culture

BMMs were isolated from whole bone marrow of six-week-old female C57BL/6 mice as described previously^42^,^43^. In brief, cells were separated from the femoral and tibial bone marrow and incubated in a T75 flask in α-MEM containing 10% FBS, 1% penicillin/streptomycin, and 30 ng/mL M-CSF for 24 h. Non-adherent cells were collected, reseeded in another T75 flask and incubated 5% CO_2_ at 37 °C for another 3–4 days until the cells reached 90% confluence. RAW264.7 cells and MDA-MB-231 cells were cultured in DMEM with 10% FBS in a humidified atmosphere of 5% CO_2_ at 37 °C. The complete medium was changed every other day. BMSCs were cultivated according to the method described by Moustafa Sayed^44^. To induce osteogenic differentiation, BMSCs were cultured in an osteogenic medium (DMEM supplemented with 10% fetal bovine serum, 50 μg/mL ascorbic acid, 10 mM glycerophosphate, 100 nM dexamethasone and 1% penicillin/streptomycin.

### *In vitro* osteoclastogenesis assay

After plating cells at a density of 8000 BMMs/well (in triplicate) into a 96-well plate and incubating for 24 h, BMMs were incubated in the presence of 30 ng/mL M-CSF, 50 ng/mL RANKL, and different concentrations of DHA (0, 1.56, 3.125, or 6.25 μM). The cell culture medium was replaced every 2 days until mature osteoclasts had formed. The cells were washed twice with phosphate-buffered saline (PBS), fixed with 4% paraformaldehyde for 20 min, and stained using the TRAP kit. TRAP-positive cells with more than three nuclei were defined as osteoclasts and counted, and the percentage of osteoclasts per well was measured using Image-Pro Plus 6.0 software.

### RNA extraction and quantitative PCR assay

Total RNA was extracted using the Qiagen RNeasy^®^ Mini kit (Qiagen, Valencia, CA, USA), and then subjected to cDNA synthesis. Real-time PCR was performed using the SYBR Premix Ex Tag kit (TaKaRa, Biotechnology, Otsu, Japan) and an ABI 7500 Sequencing Detection System (Applied Biosystems, Foster City, CA, USA) according to the manufactures’ protocols. The sequences for the relevant primers are listed in Table 1, GAPDH was used as a quantitative control gene and all reactions were run in triplicate.

### Western blotting

BMMs were seeded at a density of 2 × 10^5^ BMMs/well in 6-well plates with or without 3.125 μM DHA for 0, 1, 3, or 5 d during osteoclast induction and harvested to detect the protein expression of SRC. RAW264.7 cells were cultured to reach confluent and pretreated with or without 6.25 μM DHA for 4 h, followed by stimulation with 50 ng/mL RANKL for 0, 10, or 30 min. Cells were lysed with RIPA buffer (Beyotime, Shanghai, China) to extract proteins. Protein concentrations were determined using a bicinchoninic acid (BCA, Thermo Fisher, Waltham, MA, USA) assay. Thirty micrograms of each protein lysate were resolved using SDS-PAGE and transferred to polyvinylidene difluoride membranes (Millipore, Bedford, MA, USA). The membranes were incubated with primary antibodies at 4 °C overnight and secondary antibodies for 1 h at room temperature. Antibody reactivity was detected by exposure in an Odyssey V3.0 image scanning (Li-COR. Inc., Lincoln, NE, USA). Quantitative analysis of the band intensity was analyzed using Image J.

### Luciferase reporter gene assay

RAW264.7 cells were transfected with NFATc1 luciferase reporter constructs as described previously^45^,^46^. Briefly, cells were plated in 24-well plates at a density of 1 × 10^5^ cells/well in triplicate. After 24 h, the cells were pretreated with 0, 1.56, 3.125, or 6.25 μM DHA for 1 h, and then incubated with 50 ng/mL RANKL for 24 h to activate NFATc1. Cells were then lysed with luciferase lysis buffer, luciferase activity was detected using the Luciferase Assay Kit (Promega, Madison, WI, USA).

### F-actin Ring Immunofluorescence

The osteoclasts were fixed with 4% paraformaldehyde for 15 min at room temperature and permeabilized for 5 min with 0.1% v/v Triton X-100. The cells were then incubated with Alexa-Fluor 647 phalloidin (Invitrogen, San Diego, CA, USA) diluted in 0.2% (w/v) BSA-PBS (Invitrogen, San Diego, CA, USA) for 1 h at room temperature and washed with 0.2% w/v BSA–PBS and PBS, and DAPI was used for nuclei staining. The F-actin ring distribution was measured using the LSM5 confocal microscope (Carl Zeiss, Oberkochen, Germany). The fluorescence images were processed using the Zeiss ZEN software, and the number of intact F-actin rings was counted using Image J.

### Cell viability assay

The cytotoxic effect of DHA on BMMs or MDA-MB-231 cells were assessed using CCK-8 assays according to manufacture’s protocol. Briefly, BMMs in complete α-MEM supplemented with 30 ng/mL M-CSF were seeded in 96-well plates at a density of 8 × 10^3^ cells/well, cultured for 24 h, and treated with different concentrations of DHA for another 2, 3, 4, 5, or 6 days. MDA-MB-231 cells were cultured in 96-well plates in complete DMEM at a density of 8 × 10^3^ cells/well with increasing concentrations of DHA for 2 or 4 days. The cells were incubated with 10 μL CCK-8 buffer in each well at 37 °C for 2 h and the absorbance was measured at 450 nm (630 nm reference) on an ELX800 absorbance microplate reader (Bio-Tek, USA). Cell viability was calculated relative to that of the control cells from the optical density (OD) by using the following formula: Cell viability = (experimental group optimal density OD − zeroing OD)/(control group OD − zeroing OD).

### Transwell assay and wound healing assay

In transwell assay, Transwell® Permeable Supports and 24-well chambers with 8-μm pore polycarbonate filters were used as described by the manufacturer. MDA-MB-231 cells at a density of 5 × 10^4^ cells/well were placed in 100 μl serum-free medium in the presence or absence of different concentrations of DHA with 600 μl complete medium added into the lower wells and incubated at 37 °C for 24 h. Following treatment, cells were fixed with 100% methanol for 20 min and stained with Trypan blue for 30 min. Non-migrating cells on the upper side of the filter were removed with cotton swabs. Migration was quantified by counting the area of cells on the lower surface of the filter.

In would healing assay, a cell-based wound healing assay was performed following well-established methods^47^. Briefly, serum-starved MDA-MB-231 cells were grown to 90% confluence and a linear wound was created in the confluent monolayer using a 200 μL micropipette tip. The cells were then washed with PBS to eliminate detached cells and diluted in serum-free DMEM. Then, various concentrations of DHA were added for 12 h and 24 h of incubation, and the wound edge closure was measured with a microscope.

### Apoptosis assay

DHA induction of apoptosis in MBA-MD-231 cells was determined with the Vybrant^®^Apoptosis Assay kit #2. Following treatment, cells were washed twice with cold phosphate-buffered saline (PBS) and resuspended in 1X Annexin-binding buffer. Early apoptosis was detected via staining with Alexa Fluor^®^488 Annexin V and propidium iodide. Fluorescence-activated cell sorting was performed using a FACScan™ flow cytometer and data were acquired using Cell Quest software, version 3.0 (BD Biosciences, Sunnyvale, CA, USA).

### Histological and immunohistochemical analysis

The calvaria samples were decalcified in 10% EDTA for 3 weeks, and then embedded in paraffin. Histological sections were prepared for TRAP and H&E staining. The specimens were then examined and photographed under a high quality microscope. The number of TRAP-positive multinucleated osteoclasts was counted in each sample.

The hind limb tumor tissues were decalcified in 10% EDTA for 4 weeks. Decalcified bones were paraffin-embedded and sectioned. For histologic examination, sections were stained with hematoxylin and eosin (H&E). Immunostaining for Ki67 (Dako, Carpinteria, CA, USA) and terminal deoxynucleotidyl transferase-mediated dUTP nick-end labeling (TUNEL) were performed as previously described.

### *In vitro* bone absorption assay

BMMs were seeded at a density of 2.4 × 10^4^ cells/cm^2^ onto bovine bone slices (in triplicate), and treated with 30 ng/mL M-CSF, 50 ng/mL RANKL, and 0, 1.56, 3.125, or 6.25 μM DHA until mature osteoclasts were formed. Adherent cells were then completely removed from the slices. Resorption pits were imaged using a scanning electron microscope (SEM, FEI Quanta 250; FEI, Hillsboro, OR, USA), and the bone resorption areas were quantified using Image J software.

### Titanium-particle-induced calvarial osteolysis mice model

We established a mouse calvarial osteolysis model to measure the osteolysis-suppressing effect of DHA *in vivo*. The calvarial osteolysis mice model were develped as described previously^48^,^49^. Briefly, 24 healthy 8-week-old C57BL/J6 mice were assigned randomly into four groups: sham PBS control (sham), Ti particles with PBS (vehicle), and Ti particles with low (50 μg/kg/day) and high (100 μg/kg/day) concentrations of DHA, respectively. After abdominal anesthesia by 3.5% chloral hydrate, 30 mg of Ti particles were embedded under the periosteum at the middle suture of the calvaria. Mice in the low and high DHA groups were injected intraperitoneally with DHA at 50 or 100 μg/kg/day, respectively, for 10 days. Mice in the sham and vehicle groups received PBS daily. At the end of the experiment, the mice were sacrificed, and the calvaria of all mice were excised and fixed in 4% paraformaldehyde for micro-CT analysis.

### *In vivo* osteolytic bone metastasis assay

The MDA-MB-231 cells were cultured to reach a final concentration of 1 × 10^6^/mL and then inoculated directly into the tibiae plateau of BALB/c nu/nu mice (5–6 weeks old; female; Harlan) via a percutaneous approach. The mice were randomly assigned to 2 groups, treated with PBS (vehicle, n = 6) or DHA (100 μg/kg body weight in vehicle, n = 6) by intraperitoneal injection every other day for 28 days before sacrifice. The tibiae of all mice were scanned and analyzed with a highresolution micro-CT and the hind limb tumor tissues were proceeded with histologic examinations.

### Alkaline phosphatase (ALP) and Alizarin Red staining

BMSCs were seeded on the 24-well chambers by using the same culture conditions as those described above with 0, 1.56, 3.125, or 6.25 μM DHA. ALP and Alizarin Red staining was performed using a staining kit (Nanjing Jiancheng Chemical Industrial Co. Ltd., Nanjing, China) on days 7 and 21 of culture, respectively, according to the manufacturer’s instructions.

### Statistical analysis

The data were presented as mean ± standard deviation (SD) from at least three independent experiments. The results were analyzed using SPSS 13.0 software (IBM Corp., Armonk, NY, USA). Analysis of variance (ANOVA) with Dunnett’s post-hoc test was used for comparisons between the groups. The result of p < 0.05 and p < 0.01 indicated statistical significance between groups.

## Additional Information

**How to cite this article**: Feng, M.-X. *et al.* Dihydroartemisinin prevents breast cancer-induced osteolysis via inhibiting both breast caner cells and osteoclasts. *Sci. Rep.* **6**, 19074; doi: 10.1038/srep19074 (2016).

## Figures and Tables

**Table 1:** Sequences of primers used in real-time polymerase chain reaction (Real-time PCR).

Gene	Primer sequences (5′-3′)	
Cathepsin K	Forward	CTTCCAATACGTGCAGCAGA
	Reverse	TCTTCAGGGCTTTCTCGTTC
CTR	Forward	TGCAGACAACTCTTGGTTGG
	Reverse	TCGGTTTCTTCTCCTCTGGA
NFATc1	Forward	CCGTTGCTTCCAGAAAATAACA
	Reverse	TGTGGGATGTGAACTCGGAA
V-ATPase-d2	Forward	AAGCCTTTGTTTGACGCTGT
	Reverse	TTCGATGCCTCTGTGAGATG
V-ATPase-a3	Forward	AATCATGGACGACTCCTTGG
	Reverse	GGCCACCTCTTCACTCCGGAA
Acp 5	Forward	CACTCCCACCCTGAGATTTGT
	Reverse	CCCCAGAGACATGATGAAGTCA
GAPDH	Forward	ACCCAGAAGACTGTGGATGG
	Reverse	CACATTGGGGGTAGGAACAC
RUNX 2	Forward	GCCTTCAAGGTTGTAGCCCT
	Reverse	GGACCGTCCACTGTCACTTT
COL 1	Forward	CCCAGCGGTGGTTATGACTT
	Reverse	TCGATCCAGTACTCTCCGCT
ALP 1	Forward	CGGACAATGAGATGCGCCC
	Reverse	AGACATAGTGGGAGTGCTTGTG
Bglap	Forward	ACAGACAAGTCCCACACAGC
	Reverse	CTGGGGCTCCAAGTCCATTG
SPP 1	Forward	AGCCAGCCAAGGACCAACTA
	Reverse	TCCATGTGGTCATGGCTTTCA
Sparc	Forward	TCCTGCTGCTTGCCTCTAAA
	Reverse	ACAGGTAACCCTGTCTCCTC
GAPDH	Forward	TGGAGAAACCTGCCAAGTATGA
	Reverse	TCAGTATCCTTGCTGGGCTG
