# Corrigendum: Dihydroartemisinin prevents breast cancer-induced osteolysis via inhibiting both breast cancer cells and osteoclasts

**DOI:** 10.1038/srep46935

**Published:** 2018-01-08

**Authors:** Ming-Xuan Feng, Jian-Xin Hong, Qiang Wang, Yong-Yong Fan, Chi-Ting Yuan, Xin-Huan Lei, Min Zhu, An Qin, Hai-Xiao Chen, Dun Hong

Scientific Reports 6: Article number: 1907410.1038/srep19074; published online: 01 08 2016; updated: 01 08 2018

The original version of this Article contained an error in the title of the paper, where the word “cancer” was incorrectly given as “caner”. This has now been corrected in the PDF and HTML versions of the Article.

